# Exploring the relationship between socioeconomic factors, method of contraception and unintended pregnancy

**DOI:** 10.1186/s12978-016-0151-y

**Published:** 2016-03-22

**Authors:** Amy Metcalfe, Rachel Talavlikar, Beatrice du Prey, Suzanne C. Tough

**Affiliations:** Department of Obstetrics and Gynecology, University of Calgary, Foothills Medical Centre, 4th Floor North Tower 1403 29 St, NW Calgary, Alberta T2N 2T9 Canada; Family Medicine and Primary Care, University of Calgary, Research Office, G012, Health Sciences Centre 3330 Hospital Drive, NW Calgary, T2N 4N1 Alberta Canada; Department of Pediatrics, University of Calgary, 2888 Shaganappi Trail, N.W. Calgary, Alberta T3B 6A8 Canada; Department of Community Health Sciences, University of Calgary, Calgary, Alberta T2N Canada

**Keywords:** Contraception, Unintended pregnancy, Socio-economic status, Pregnancy

## Abstract

**Background:**

It is estimated that approximately one-third of pregnancies in Canada are unintended, meaning they were either mistimed (the woman wanted to be pregnant at a different point in time) or undesired (the woman did not want to be pregnant). This study aimed to assess the impact of socioeconomic variables and method of contraception on the decision to either terminate or continue and unintended pregnancy.

**Methods:**

Data were obtained from two contemporaneous studies in Calgary Canada– a cross-sectional study involving women seeking abortion services (*n* = 577) and a longitudinal cohort study involving women with continuing pregnancies (*n* = 3552) between 2008 and 2012. Chi square tests and logistic regression were used to examine the association between socioeconomic variables, use of contraception and pregnancy intention.

**Results:**

96.5 % of women seeking an abortion and 19.6 % of women with ongoing pregnancies reported having an unintended pregnancy. Women with unintended pregnancies were significantly younger (*p* < 0.001), less educated (*p* < 0.001), had a lower household income (*p* < 0.001), were less likely to be in a stable relationship (*p* < 0.001), and less likely to speak English in the home (*p* < 0.002). 20.2 % reported not using any form of birth control despite their desire to not get pregnant. Among women with unintended pregnancies, the only significant demographic predictor of not using any form of contraception was low educational attainment (OR = 1.7, 95 % CI: 1.2–2.4).

**Conclusions:**

Low educational attainment was associated with not using any form of contraception among women with unintended pregnancies. However, as unintended pregnancy occurs across all socio-demographic groups, care providers are encouraged to have an open discussion regarding fertility goals and contraception with all patients and refer them to appropriate resource materials.

## Background

It is estimated that approximately one third of pregnancies in Canada are unintended, meaning they were either mistimed (the woman wanted to be pregnant at a different point in time) or undesired (the woman did not want to be pregnant) [1]. While approximately half of unintended pregnancies end in termination [2]; unintended does not necessarily mean unwanted [3], and a proportion of these pregnancies result in live births. Current literature supports that for women who choose to continue an unintended pregnancy, there is an association between pregnancy intention and a variety of unhealthy behaviours during pregnancy such as smoking and late initiation of prenatal care, as well as adverse pregnancy outcomes such as post-partum depression, low birth weight and preterm birth [4–6].

Socioeconomic factors influence both choice and use of contraception, the rate of unintended pregnancy, and the decision to continue or terminate an unintended pregnancy [1, 7–10], resulting in a complex relationship between all of these factors and reproductive decision-making. Additionally, different forms of contraception have varying efficacy [1, 11]. A 2006 national Canadian survey found that among those sampled, 15 % of sexually active women had never used contraception and less than 5 % of women had used long acting reversible forms of contraception with the lowest failure rates such as intrauterine devices or injections [1].

While many studies have described contraception usage and socioeconomic variables amongst women seeking abortions or women with unintended continuing pregnancies; no one has simultaneously examined these groups to determine if these factors impact women’s decisions about whether to continue or terminate an unintended pregnancy. This study aimed to assess the impact of socioeconomic variables and method of contraception on the decision to either terminate or continue and unintended pregnancy.

An examination of measures to prevent unintended pregnancies in the Canadian context is important from a health policy perspective as while health insurance is publicly available for Canadian citizens and permanent residents, public health care insurance does not extend to include the cost of the contraception itself) [12]. Women wishing to assess contraception in Canada must either pay out of pocket or have additional private health insurance. Even for women who have access to subsidized health insurance plans through government benefit programs or private insurance, often items such as copper intrauterine devices (IUDs) are not accessible as they are considered medical devices, not drugs [12]. As with many health issues, it is much more cost effective to focus on prevention rather than management within a health system. By understanding if certain population groups are (and are not) effectively using contraception, public health interventions can be more appropriately targeted to promote contraceptive use in groups at higher risk for unintended pregnancies.

## Methods

Two pregnancy-related studies were conducted simultaneously with pregnant women in Calgary Alberta – the “All Our Babies” study and the “Induced Abortion and Contraception Use in Immigrant and Canadian-Born Women in Calgary” study. The “All Our Babies” study was a community-based prospective longitudinal cohort of women’s experiences during pregnancy and the post-partum period and involved women with intended and unintended pregnancies who had elected to continue the pregnancy [13]. Women were recruited from the community between 2008 and 2010 and were initially asked to complete two questionnaires during pregnancy (<24 weeks of gestation and 34–36 weeks of gestation) and one at four months postpartum [13]. Additional follow-up questionnaires were sent at 1, 2, 3 and 5 years postpartum [13]. Of 4011 women who were assessed for eligibility 3388 (84.5 %) women completed at least one survey [13]. Women were eligible to participate in the “All Our Babies” study if they were 18 years of age or older, were ≤24 weeks gestational age at the time of recruitment, were receiving prenatal care in Calgary and were able to complete the written questionnaires in English [13]. The “Induced Abortion and Contraception Use in Immigrant and Canadian-Born Women in Calgary” study was a cross-sectional study of 752 women (response rate 78.6 %) seeking abortions in Calgary [14]. Women were recruited at local abortion clinics between 2011 and 2012 and were asked to complete a single questionnaire [14]. Women were eligible to participate if they were <15 weeks of gestation at the time of recruitment and were receiving an elective abortion in Calgary [14]. Both studies collected similar data on socioeconomic variables and use of contraception. Key variables of interest for this study were maternal age, educational attainment, household income, marital status, language spoken in the home, and type of contraception used. De-identified data from both studies was combined to create a new study cohort. Full details on individual cohort recruitment and eligibility criteria can be found elsewhere [13, 14]. Ethical approval was obtained from the Conjoint Health Research Ethics Board at the University of Calgary to conduct the original studies and to conduct this secondary analysis.

Descriptive statistics were used to characterize the population. Chi-square tests were used to assess differences in categorical outcomes amongst women with unintended pregnancies. Logistic regression was used to examine the association between socio-demographic factors and the following outcome variables: having an unintended pregnancy, pregnancy termination following an unintended pregnancy and use of contraception prior to an unintended pregnancy. Use of contraception was defined as using any form of contraception and using multiple forms of contraception. Multiple forms of contraception referred to using at least two methods of contraception, although they may be of similar efficacy and neither may protect against sexually transmitted diseases (e.g. a women using both the rhythm method and the birth control pill or condoms and a cervical cap were classified as using multiple forms of contraception). All covariates (educational attainment, language spoken at home, household income, marital status and age) were included in all models. Alpha <0.05 was used to define statistical significance. For women with unintended pregnancies (the primary group of interest in this study), missing data was minimal for all variables of interest (i.e. less than five respondents for all variables except for income where missing data was present for 54 (4.5 % of respondents) and was addressed using list-wise deletion.

## Results

Overall, 3929 women completed at least one survey and provided data on pregnancy intention, while 1211 women (30.8 %, 95 % CI: 29.4–32.3) reported that their pregnancies were unintended; representing 19.6 % (95 % CI: 18.3–21.0, *n* = 657) of women in the All Our Babies cohort and 96.5 % (95 % CI: 94.7–97.7, *n* = 554) of women seeking abortion. For women in the All Our Babies cohort, 20.4 % (95 % CI: 19.0–21.7) of pregnancies were mistimed and 2.6 % (95 % CI: 2.1–3.2) were unwanted. In the combined cohort, compared to women with planned pregnancies, women who had an unintended pregnancy were younger, had lower educational attainment, lower household income and were more likely to be single, divorced or widowed (Table 1). Among women with unintended pregnancies, women who had an abortion compared to women who continued their pregnancy were more likely to have lower educational attainment, lower household income and were more likely to be single, divorced or widowed; however, women in the oldest age category were the most likely to have an abortion (Table 2).

**Table 1:** Socio-demographic pedictors of unintended pregnancy (*n* = 3929)

Variable	Women with planned pregnancies	Women with unintended pregnancies	Adjusted odds ratio (95 % confidence interval)
	*N* (%, 95 % CI)	*N* (%, 95 % CI)	
Highest level of education completed			
Post-secondary	2479 (91.3, 90.2–92.3)	817 (67.6, 64.9–70.2)	Ref
High school	235 (8.7, 7.7–9.8)	391 (32.4, 29.8–35.1)	**2.1 (1.7**–**2.7)**
Speaks English in the home			
Yes	2395 (88.2, 87.0–89.4)	1023 (84.6, 82.5–86.5)	Ref
No	319 (11.8, 10.6–13.0)	186 (15.4, 13.5–17.5)	**1.3 (1.0**–**1.7)**
Low income (household income < $42,000/year)			
No	2443 (92.9, 91.9–93.8)	726 (62.7, 59.9–65.5)	Ref
Yes	186 (7.1, 6.2–8.1)	431 (37.3, 34.5–40.1)	**2.84 (2.2**–**3.6)**
Marital status			
Married/common law	2650 (97.8, 97.2–98.3)	778 (64.3, 61.6–67.0)	Ref
Single/divorced/widowed	60 (2.2, 1.7–2.8)	432 (35.7, 33.0–38.4)	**13.6 (10.0**–**18.4)**
Maternal age			
≥ 40 years	74 (2.8, 2.2–3.5)	27 (2.3, 1.5–3.3)	Ref
35–39 years	469 (17.7, 16.3–19.2)	161 (13.4, 11.6–15.5)	1.2 (0.7–2.1)
30–34 years	1121 (42.3, 40.4–44.2)	289 (24.1, 21.8–26.6)	1.0 (0.6–1.8)
25–29 years	829 (31.3, 29.5–33.1)	338 (28.2, 25.7–30.8)	1.3 (0.8–2.2)
20–24 years	150 (5.7, 4.8–6.6)	280 (23.4, 21.0–25.8)	**2.9 (1.6**–**5.1)**
< 20 years	7 (0.3, 0.1–0.6)	104 (8.7, 7.2–10.4)	**8.0 (2.8**–**22.8)**

Bolded text indicates statistically significant differences (*p* < 0.05)

Pseudo *R* ^2^ = 0.24

Adjusted odds ratios were obtained through a multivariate logistic regression model that adjusted for educational attainment, language spoken in the home, household income, marital status, and maternal age

**Table 2:** Socio-demographic predictors of pregnancy termination following an unintended pregnancy (*n* = 1211)

Variable	Women who continued their pregnancy	Women who terminated their pregnancy	Adjusted odds ratio (95 % confidence interval)
	*N* (%, 95 % CI)	*N* (%, 95 % CI)	
Highest level of education completed			
Post-secondary	514 (78.6, 75.3–81.6)	303 (54.7, 50.5–58.8)	Ref
High school	140 (21.4, 18.4–24.7)	251 (45.3, 41.2–49.5)	**1.9 (1.4**–**2.59)**
Speaks English in the home			
Yes	578 (88.0, 85.3–90.3)	445 (80.6, 77.1–83.7)	Ref
No	79 (12.0, 9.7–14.7)	107 (19.4, 16.3–22.9)	**1.9 (1.3**–**2.77)**
Low income (household income < $42,000/year)			
No	512 (80.9, 77.6–83.8)	214 (40.8, 36.7–45.1)	Ref
Yes	121 (19.1, 16.2–22.4)	310 (59.2, 54.9–63.3)	**3.8 (2.9**–**5.17)**
Marital status			
Married/common law	517 (78.8, 75.5–81.8)	261 (47.1, 43.0–51.3)	Ref
Single/divorced/widowed	139 (21.2, 18.2–24.5)	293 (52.9, 48.7–57.0)	**3.1 (2.3**–**4.18)**
Maternal age			
≥ 40 years	7 (1.1, 0.5–2.3)	20 (3.6, 2.3–5.5)	Ref
35–39 years	96 (14.9, 12.3–17.9)	65 (11.7, 9.3–14.7)	**0.3 (0.1**–**0.7)**
30–34 years	205 (31.8, 28.3–35.5)	84 (15.2, 12.4–18.4)	**0.2 (0.1**–**0.4)**
25–29 years	196 (30.4, 27.0–34.1)	142 (25.6, 22.2–29.4)	**0.2 (0.1**–**0.6)**
20–24 years	115 (17.8, 15.1–21.0)	165 (29.8, 26.1–33.7)	**0.2 (0.1**–**0.6)**
< 20 years	26 (4.0, 2.8–5.9)	78 (14.1, 11.4–17.2)	**0.3 (0.1**–**0.7)**

Bolded text indicates statistically significant differences (*p* < 0.05)

Pseudo *R* ^2^ = 0.20

Adjusted odds ratios were obtained through a multivariate logistic regression model that adjusted for educational attainment, language spoken in the home, household income, marital status, and maternal age

Among women with unintended pregnancies, 20.1 % (95 % CI: 18.0–22.5) were not using any form of contraception. The only significant demographic predictor of not using any form of contraception was low educational attainment (OR = 1.7, 95 % CI: 1.2–2.4) (Table 3). Additionally, the proportion of women with unintended pregnancies who had not been using any form of contraception was significantly higher in women who ultimately terminated the pregnancy (23.7 %, 95 % CI: 20.3–27.4) than those who continued the pregnancy (17.2 %, 95 % CI: 14.5–20.3) (*p* = 0.005).

**Table 3:** Socio-demographic predictors of failure to use contraception amongst women with unintended pregnancies (*n* = 1211)

Variable	Women who were using contraception	Women who were not using contraception	Adjusted odds ratio (95 % confidence interval)
	**N (%, 95 % CI)**	**N (%, 95 % CI)**	
Highest level of education completed			
Post-secondary	678 (70.5, 67.5–73.3)	139 (57.7, 51.3–63.8)	Ref
High school	284 (29.5, 26.7–32.5)	102 (42.3, 36.2–48.7)	**1.7 (1.2**–**2.4)**
Speaks English in the home			
Yes	810 (84.2, 81.8–86.4)	210 (86.4, 81.5–90.2)	Ref
No	152 (15.8, 13.6–18.2)	33 (13.6, 9.8–18.5)	0.8 (0.5–1.2)
Low income (household income < $42,000/year)			
No	590 (64.1, 61.0–67.2)	134 (57.8, 51.3–64.0)	Ref
Yes	330 (35.9, 32.8–39.0)	98 (42.2, 36.0–48.7)	1.3 (0.9–1.8)
Marital status			
Married/common law	623 (64.8, 61.7–67.7)	151 (62.1, 55.8–68.1)	Ref
Single/divorced/widowed	339 (35.2, 32.3–38.3)	92 (37.9, 31.9–44.2)	1.0 (0.7–1.4)
Maternal age			
≥ 40 years	21 (2.2, 1.4–3.4)	6 (2.5, 1.1–5.5)	Ref
35–39 years	122 (12.8, 10.8–15.1)	36 (15.1, 11.0–20.2)	1.0 (0.4–2.6)
30–34 years	234 (24.5, 21.9–27.3)	55 (23.0, 18.1–28.8)	0.8 (0.3–2.0)
25–29 years	280 (29.3, 26.5–32.3)	57 (23.8, 18.8–29.7)	0.6 (0.2–1.5)
20–24 years	225 (23.6, 21.0–26.4)	54 (22.6, 17.7–28.4)	0.6 (0.2–1.5)
< 20 years	73 (7.6, 6.1–9.5)	31 (13.0, 9.2–17.9)	0.8 (0.3–2.4)

Bolded text indicates statistically significant differences (*p* < 0.05)

Pseudo *R* ^2^ = 0.02

Adjusted odds ratios were obtained through a multivariate logistic regression model that adjusted for educational attainment, language spoken in the home, household income, marital status, and maternal age

Barrier (33.1 %, 95 % CI: 30.3–35.9) and hormonal (27.7 %, 95 % CI: 25.1–30.4) methods were the most common forms of contraception used by women in the combined cohort (Table 4). Socio-demographic factors influenced not only the use of contraception, but also the type of contraception used (Table 4). While women with lower educational attainment tended to use barrier or hormonal methods of conception, they were also significantly more likely than women with more education to have not used any form of contraception prior to an unplanned pregnancy (28.0 % vs. 19.6 %). Women who did not speak English in the home were significantly more likely (45.1 % vs. 30.9 %) to have used barrier methods and significantly less likely (13.4 % vs. 30.2 %) to have used hormonal methods compared to women whose primary language at home was English. While not reaching statistical significance, women who were married or in a common-law relationship were also more likely to be using natural family planning methods (12.1 % vs. 7.4 %) and surgical methods (4.2 % vs. 2.5 %) compared to women in a less stable relationships. While not always achieving statistical significance, a clear linear relationship was observed between maternal age and choice of contraceptive method – a higher proportion of older women used natural family planning methods and barrier methods, while younger women were significantly more likely to use hormonal methods. Like all women in this cohort with unintended pregnancies, women who opted to terminate tended to use barrier and hormonal methods, but were also significantly more likely to have used emergency contraception (4.6 % vs. 1.1 %). No statistically significant differences were observed by income status with regards to type of contraception used.

**Table 4:** Bivariate association between socio-demographic factors, decision regarding pregnancy termination, and most effective type of contraception amongst women with unintended pregnancies (*n* = 1211)

	Most effective type of contraception used					
	None	Natural Family planning	Barrier methods	Hormonal methods	Emergency contraception	Surgical methods
	% (95 % CI)	% (95 % CI)	% (95 % CI)	% (95 % CI)	% (95 % CI)	% (95 % CI)
Overall	22.6 (20.2–25.2)	10.3 (8.6–12.3)	33.1 (30.3–35.9)	27.7 (25.1–30.4)	2.9 (2.0–4.1)	3.5 (2.6–4.8)
Highest level of education completed						
Post-secondary	**19.6 (16.8**–**22.7)**	**12.5 (10.3**–**15.2)**	34.2 (30.8–37.8)	26.3 (23.2–29.7)	3.5 (2.4–5.2)	3.8 (2.6–5.5)
High school	**28.0 (23.6**–**32.9)**	**6.0 (4.0**–**9.0)**	30.8 (26.2–35.7)	30.5 (26.0–35.4)	1.6 (0.7–3.6)	3.0 (1.7–5.4)
Speak English in the home						
Yes	23.0 (20.4–25.9)	10.2 (8.4–12.3)	**30.9 (28.0**–**34.0)**	**30.2 (27.3**–**33.2)**	2.6 (1.8–3.9)	3.1 (2.1–4.4)
No	20.1 (14.7–27.0)	11.0 (7.0–16.8)	**45.1 (37.6**–**52.8)**	**13.4 (9.0**–**19.6)**	4.3 (2.0–8.7)	6.1 (3.3–11.0)
Low income (household income < $42,000/year)						
No	21.2 (18.2–24.5)	12.2 (9.8–15.0)	33.8 (30.2–37.6)	28.1 (24.7–31.8)	2.1 (1.2–3.5)	2.7 (1.7–4.3)
Yes	24.7 (20.7–29.2)	7.1 (4.9–10.0)	33.0 (28.5–37.8)	27.2 (23.0–31.8)	3.8 (2.3–6.2)	4.3 (2.7–6.8)
Marital status						
Married/common law	22.5 (19.5–25.8)	12.1 (9.8–14.7)	33.0 (29.6–36.7)	27.2 (24.0–30.7)	**1.0 (0.5**–**2.2)**	4.2 (2.9–6.0)
Single/divorced/widowed	22.8 (18.9–27.1)	7.4 (5.2–10.4)	33.2 (28.7–37.9)	28.2 (24.0–32.8)	**5.9 (4.0**–**8.7)**	2.5 (1.3–4.5)
Maternal age						
≥ 40 years	23.1 (10.6–43.2)	15.4 (5.8–35.0)	38.5 (21.8–58.3)	**7.7 (1.9**–**26.7)**	0	**15.4 (5.8**–**35.0)**
35–39 years	26.7 (19.9–34.8)	14.1 (9.1–21.1)	35.6 (27.9–44.0)	**17.0 (11.6**–**24.4)**	3.0 (1.1–7.7)	3.7 (1.5–8.6)
30–34 years	22.2 (17.4–27.8)	14.1 (10.3–19.0)	37.9 (32.1–44.1)	**19.0 (14.5**–**24.3)**	2.4 (1.1–5.3)	4.4 (2.5–7.8)
25–29 years	18.8 (14.8–23.6)	9.2 (6.4–13.1)	32.7 (27.6–38.2)	31.7 (26.7–37.2)	3.3 (1.8–6.0)	4.3 (2.5–7.3)
20–24 years	21.0 (16.5–26.5)	6.6 (4.2–10.4)	29.3 (24.0–35.2)	**37.5 (31.8**–**43.6)**	3.5 (1.8–6.6)	**2.0 (0.8**–**4.6)**
< 20 years	31.3 (22.9–41.1)	7.0 (3.4–14.2)	27.3 (19.4–36.9)	32.3 (23.8–42.2)	2.0 (0.5–7.8)	0
Decision following an unintended pregnancy						
Pregnancy termination	24.0 (20.6–27.8)	**6.1 (4.4**–**8.4)**	34.7 (30.8–38.8)	26.0 (22.5–29.9)	**4.6 (3.1**–**6.7)**	4.6 (3.1–6.7)
Pregnancy continuation	21.1 (17.9–24.8)	**14.6 (11.8**–**17.8)**	31.4 (27.6–35.5)	29.3 (25.6–33.4)	**1.1 (0.5**–**2.5)**	2.4 (1.4–4.1)

Table Notes: Natural family planning includes withdrawal and the rhythm method; barrier methods include condoms, cervical caps and spermicides; hormonal methods include injectables, the birth control patch, birth control pills and the birth control ring; surgical methods include intrauterine devices, tubal ligation and vasectomies. Intrauterine devices were classified as a surgical method as they are inserted by a health care provider

Bolded text indicates statistically significant differences (*p* < 0.05)

Chi square tests were used to assess statistical differences between groups

Overall, 224 women (18.5 %, 95 % CI: 16.4–20.8) with unintended pregnancies reported using multiple forms of contraception prior to an unintended pregnancy. Women who opted to continue (18.3 %, 95 % CI: 15.5–21.4) or terminate (18.8 %, 95 % CI: 15.7–22.2) an unintended pregnancy were equally likely to be using multiple forms of birth control. Amongst women using some form of birth control, women who did not speak English in the home (OR = 0.8, 95 % CI: 0.6–1.0, *p* = 0.03) were significantly less likely to have been using multiple forms of contraception prior to an unintended pregnancy (Table 5). Amongst women who reported using multiple forms of contraception prior to an unintended pregnancy, 14.7 % (95 % CI: 10.6–20.0) combined two methods with similar efficacy (i.e. withdrawal and natural family planning), while 85.3 % (95 % CI: 80.0–89.4) combined a less effective method (i.e. condoms) with a more effective method (i.e. hormonal). This was not significantly associated with any socio-demographic variables or decisions regarding pregnancy termination.

**Table 5:** Socio-demographic predictors of using multiple forms of contraception prior to an unintended pregnancy amongst women using some form of contraception (*n* = 963)

Variable	Women using a single form of contraception	Women using multiple forms of contraception	Adjusted odds ratio (95 % confidence interval)
	*N* (%, 95 % CI)	*N* (%, 95 % CI)	
Highest level of education completed			
Post-secondary High school	520 (70.5, 67.1–73.6) 218 (29.5, 26.4–32.9)	158 (70.5, 64.2–76.2) 66 (29.5, 23.8–35.8)	Ref 0.9 (0.6–1.1)
Speaks English in the home			
Yes No	617 (83.6, 80.7–86.1) 121 (16.4, 13.9–19.3)	193 (86.2, 80.9–90.1) 31 (13.8, 9.9–19.1)	Ref **0.8 (0.6**–**1.0)**
Low income (household income < $42,000/year)			
No Yes	460 (65.3, 61.7–68.8) 244 (34.7, 31.2–38.3)	130 (60.2, 53.5–66.5) 86 (39.8, 33.5–46.5)	Ref 1.4 (1.0–1.8)
Marital status			
Married/common law Single/divorced/widowed	486 (65.9, 62.3–69.2) 252 (34.1, 30.8–37.7)	137 (61.2, 54.6–67.4) 87 (38.8, 32.6–45.4)	Ref 1.2 (0.9–1.6)
Maternal age			
≥ 40 years 35–39 years 30–34 years 25–29 years 20–24 years < 20 years	14 (1.9, 1.1–3.2) 97 (13.2, 11.0–15.9) 189 (25.8, 22.7–29.1) 209 (28.5, 25.4–31.9) 166 (22.6, 19.8–25.8) 58 (7.9, 6.2–10.1)	7 (3.2, 1.5–6.5) 25 (11.3, 7.7–16.2) 45 (20.3, 15.5–26.1) 71 (32.0, 26.1–38.5) 59 (26.6, 21.1–32.8) 15 (6.8, 4.1–10.9)	Ref 0.9 (0.5–1.8) 1.1 (0.6–2.0) 1.2 (0.7–2.3) 1.3 (0.6–2.5) 0.8 (0.3–2.0)

Bolded text indicates statistically significant differences (*p* < 0.05)

Pseudo *R* ^*2*^ = 0.01

Multiple forms of contraception refers to the use of two more types of contraception, even if both methods used had similar efficacy

Adjusted odds ratios were obtained through a multivariate logistic regression model that adjusted for educational attainment, language spoken in the home, household income, marital status, and maternal age

## Discussion

Unintended pregnancy is a common occurrence and can result from both a failure to use contraception and contraception failure. This study of women with unintended pregnancies shows that both use of contraception overall, choice of contraception and pregnancy termination following an unintended pregnancy are related to socio-demographic factors; however, as unintended pregnancy occurs across all social groups, education in this area should not be restricted to targeted populations. The pseudo R^2^ values for each of the models in this study were low, indicating that at best, socio-demographic factors can explain approximately 20 % of unintended pregnancies and women’s choices regarding pregnancy continuation or termination following an unintended pregnancy. The pseudo R^2^ values also show that socio-demographic variables can only explain approximately 1 % of the variance in use of contraception. Use of contraception is a complex issue that is more likely influenced by relationship factors than socio-demographic characteristics.

Few differences were observed in which type of contraception was used between women who continued and terminated an unintended pregnancy; however, women who ultimately terminated an unintended pregnancy were significantly more likely to have used emergency contraception. However, the low utilization of emergency contraception overall may indicate that women have difficulty accessing this important form of contraception or low knowledge levels about its safety and efficacy. A 2012 survey of sexually active European women found that 33 % of women did not understand how emergency contraception works and 46 % of women did not understand that they might get pregnant after unprotected intercourse [15]. Canadian data suggests that there may also be less availability of emergency contraception in rural areas due to restricted pharmacy hours (i.e. 15 % are closed on weekends) [16]. Cultural beliefs may also influence the type of contraception used [17]. In this study, women who did not speak English in the home were significantly more likely to use barrier methods and significantly less likely to use hormonal methods of contraception. Barrier methods may be perceived by some as being more ‘natural’ and in line with religious or cultural teachings. However, as there is little information on the impact of traditional cultural teachings on contraception usage for women who have immigrated to North America, care providers are cautioned to not attribute stereotypical beliefs to women from different cultures who are at risk for unintended pregnancies [17].

In this sample of women with unintended pregnancies, approximately 20 % of women reported not using any form of contraception. This is comparable to a national survey of current contraception use, conducted in Canada in 2006, which found that amongst sexually active women who were not currently trying to conceive, 15 % never used contraception [1]. Failure to use contraception consistently and correctly is a complex issue related to knowledge, access and relationship characteristics [9]. In this study, income was not associated with the use of contraception overall or any specific contraceptive method. This may indicate that affordability of contraception is not the primary barrier to use in this setting.

In both univariable and multivariable analyses, low educational attainment was the only variable significantly associated with failure to use contraception in this study. It is plausible that more consistent use of contraceptive counselling in primary care may help address this barrier for women with lower educational attainment. A survey of women attending four primary care clinics in the US found that contraceptive counselling was not consistently provided to all women accessing primary care, and women who had received contraceptive counselling at their last visit were typically younger, single and more likely to have previously been pregnant [18]. However, women who had merely discussed contraception with their health provider at their last visit were significantly more likely to have used some form of reversible contraception during their last sexual encounter (OR = 2.36, 95 % CI: 1.49–3.74) [18]. A recent randomized clinical trial found that referring women to a Facebook page with accurate information on contraceptive options (compared to providing them with a pamphlet) in addition to standard in-office contraceptive counselling was associated with improved knowledge (*p* < 0.001) and satisfaction (*p* < 0.001) [19]. The use of social media may be a novel way to engage large groups of women and provide them with accurate and timely information about their contraceptive options.

This study is not without limitations as it is based on self-reported data from women who agreed to participate in pregnancy-related research. Both surveys had high response rates [13, 14] and women who participated in the All Our Babies cohort are reflective of the pregnant and parenting population in Canada [13]. While minimal, missing data did occur, particularly in relation to household income. Pregnancy intentions are not necessarily a fixed entity and even though a pregnancy is unintended, that does not necessarily mean that it is unwanted [3, 20]. In this study pregnancy intention was only assessed at a single time point in the first or second trimester. We were also unable to identify if woman in the All Our Babies cohort unintended pregnancies and later had spontaneous or induced abortions. Women were included in the present study if they completed at the first questionnaire in this longitudinal cohort study, regardless of pregnancy outcome. Additionally, no data was collected on women’s reasons for not using contraception or their knowledge level with regards to contraception or fertility in the All Our Babies cohort (although this data was available in the abortion cohort and results can be found elsewhere [14]).

## Conclusions

In conclusion, low educational attainment was associated with not using any form of contraception among women with unintended pregnancies. This speaks to the need to further integrate education about fertility and contraception into the public school system before young people become sexually active and to reinforce this message in multiple forms as people progress through the school system to ensure increased knowledge. As unintended pregnancies occur across all socio-demographic groups, care providers are encouraged to have an open discussion regarding fertility goals and contraception with all patients and refer them to appropriate resource materials.
